# Survey-identified experiences of prediagnosis and diagnosis process among patients with COPD, asthma, interstitial lung disease and bronchiectasis

**DOI:** 10.1136/bmjresp-2022-001588

**Published:** 2023-11-22

**Authors:** Xiubin Zhang, Andrew Ellis, Jennifer K Quint, Alex Bottle

**Affiliations:** 1 School of Public Health, Imperial College, London, UK; 2 Asthma and Lung UK, London, UK

**Keywords:** COPD, asthma, interstitial lung disease, bronchiectasis, diagnosis, online survey

## Abstract

**Introduction:**

Diagnosis of asthma, chronic obstructive pulmonary disease (COPD), bronchiectasis and interstitial lung disease (ILD) can be convoluted, and limited data exist on understanding the experience of diagnosis from a patient perspective.

**Aim:**

To investigate a patient’s ‘route to diagnosis’, particularly focusing on the time prior to seeking healthcare, and perceived experiences of the diagnostic pathway.

**Methods:**

An online survey was distributed via the UK Taskforce for Lung Health and member mailing lists to patients as well as the website and social media accounts from 23 May 2022 to 5 July 2022. Analysis was descriptive; χ^2^ tests were performed to make comparisons across diseases.

**Results:**

There were 398 valid responses (COPD=156, asthma=119, ILD=67 and bronchiectasis=56). While only 9.2% of respondents who were eventually diagnosed with asthma had not heard of their disease, the corresponding percentages for COPD, ILD and bronchiectasis were 34.0%, 74.6% and 69.6%, respectively. 33.9% of people with bronchiectasis believed their delayed diagnosis was due to the health professionals’ lack of expertise or knowledge—24.4% for asthma, 19.2% for COPD and 17.9% for ILD.

People with COPD were more likely (37.2%) and patients with asthma less likely (10.9%) to report they did not know the signs of potential lung disease (p<0.001). People with COPD were more likely to report that they did not appreciate the severity or urgency of the situation (58.3%) than people with asthma (32.8%), ILD (43.3%) or bronchiectasis (28.6%, p<0.001). The proportion of patients reporting that they were being initially treated for another lung condition was higher in people with bronchiectasis (44.6%) and lower in people with asthma (8.4%, p<0.001).

**Conclusions:**

Perceived reasons for diagnostic delay can help health professionals promote early diagnosis and management. Patients’ limited knowledge of respiratory diseases also played a factor, indicating the necessity to promote patients’ knowledge to encourage earlier help seeking.

**WHAT IS ALREADY KNOWN ON THIS TOPIC**

- The barriers and facilitators of pathways to diagnosis have been widely studied. However, there is little information as to what extent England’s National Health Service (NHS) is meeting the needs of current patients with respiratory conditions from the patient’s perspective.

**WHAT THIS STUDY ADDS**

- The study identified some perceived experiences and barriers of diagnostic delay in patients with respiratory diseases. Reasons for delayed diagnosis were mainly due to healthcare professionals limited awareness and patients lacking the knowledge to recognise symptoms.

**HOW THIS STUDY MIGHT AFFECT RESEARCH, PRACTICE OR POLICY**

- This study highlights the necessity of promoting patients’ knowledge of their diseases and helps healthcare professionals understand respiratory patients’ experience of the diagnosis process to improve early diagnosis and management.

## Introduction

Chronic respiratory diseases are the third leading cause of morbidity and mortality worldwide, of which chronic obstructive pulmonary disease (COPD), asthma, interstitial lung disease (ILD) and bronchiectasis are the most prevalent chronic respiratory diseases.^1^ Morbidity and mortality associated with these diseases are increasing with the global ageing population.^2^ A systematic review reported that there were 544.9 million people worldwide who had a chronic respiratory disease in 2017, an increase of almost 40% compared with 1990.^1^ Globally, 3.2 million deaths were due to COPD and 495 000 deaths were due to asthma in 2017.^3^

Accurate and timely diagnosis is key for respiratory disease management and preventing deterioration. Previous studies in people with COPD have indicated that early diagnosis and intervention reduces hospitalisation, morbidity and mortality associated with the disease.^4 5^ A recent adult breathlessness diagnostic pathway support tool by National Health Service (NHS) England reported that 58% of patients with COPD presented with respiratory symptoms up to 5 years prior to diagnosis.^6^ Early intervention and management after diagnosis are important in disease control, especially self-management in people with long-term conditions. Previous studies have reported that self-management educational programmes that focus on acquisition of self-management skills and behavioural change reduce the need for rescue medication in people with chronic respiratory diseases.^7^ One previous qualitative study indicated that if integrated care was coordinated under the local hospital, this would benefit self-care for the patients.^8^

To improve health outcomes, management of disease ideally needs to be tailored to each patient, so understanding patients’ perception of their health condition and diagnostic processes are very important with respect to clinical decision-making. Understanding pathways to diagnosis not only helps to identify barriers and facilitators of early diagnosis, it also creates an opportunity to develop universal cost-effective interventions and therapeutic support in clinical settings.^5^ However, there is little information as to the extent to which England’s NHS is meeting the needs of current patients with respiratory conditions and how well it might meet the needs of future patients. In this study, we invited people with a diagnosis of COPD, asthma, ILD or bronchiectasis to participate in an online survey. We asked how patients obtained their diagnosis (the ‘route to diagnosis’), particularly focusing on the time prior to seeking healthcare so that we could understand the reasons that brought individuals to seek healthcare support. We also explored perceived experiences of both the time prediagnosis and the process of diagnosis, comparing and contrasting among people with COPD, asthma, ILD or bronchiectasis.

## Methods

### Study design

A cross-sectional survey (online supplemental appendix 1) was designed jointly by researchers and patient representatives at Imperial College London and the teams at Asthma+Lung UK and the Taskforce for Lung Health (TFLH). Survey questions were developed through a series of discussions with experts (n=10) in the field (including experts from the research team (5), a small number of physicians (2), TFLH and the Asthma+Lung UK patient advisory group (3)). The draft survey was piloted with the patient advisory group with one round of feedback; this piloting highlighted wording issues for correction and estimated the total time taken to complete the survey.

10.1136/bmjresp-2022-001588.supp1 Supplementary data

The survey questions were converted into an online format (Survey Monkey) and distributed via the TFLH members mailing lists as well as its website and social media accounts from 23 May 2022 to 5 July 2022. Patients living in England and using NHS with a diagnosis of COPD, asthma, ILD or bronchiectasis were invited to complete the survey. To be qualified for entry into the survey, respondents were required to meet the following criteria: (1) consent to participate the survey, (2) understand survey confidentiality, (3) have a diagnosis of COPD, asthma, ILD or bronchiectasis and (4) currently live in England. If respondents did not meet these criteria, they were unable to access the survey further or their data were excluded.

### Ethical approvals

We have Health Research Authority (HRA) and Health and Care Research Wales (HCRW) Approval for this study, REC reference 21/PR/0098.

### Patients and public involvement

Two patient representatives in the study team at Imperial College London and the teams at Asthma+Lung UK and the TFLH contributed throughout the duration of the study. They commented on the initial design of the study and the online survey questions. In addition, they corrected wording issues and estimated the total time taken to complete the survey. Finally, one study team patient representative commented on the study findings.

### Data analysis

Data analysis was predominantly descriptive by reporting proportions. χ^2^ tests were performed using SPSS V.26 to observe the categorical variables and make comparisons across the four diseases (online supplemental appendix 2). For all tests, p≤0.05 was considered statistically significant. Thematic analysis approach was applied to analyse the questions answered in free text, and the qualitative software NVivo V.20 was used to help with data management and analysis.

## Results

### Characteristics of the participants

A total of 428 individuals (COPD=171, asthma=129, ILD=78, bronchiectasis=60) entered data into the survey. After eliminating disqualified responses, there were 398 valid responses (COPD=156, asthma=119, ILD=67 and bronchiectasis=56). There were 79.5% (n=124) female COPD respondents, 58.8% (n=70) female asthma respondents, 58.2% (n=39) female ILD respondents and 58.8% (n=37) females bronchiectasis respondents (table 1).

**Table 1:** Characteristics of respondents

Characteristics of respondents		Asthma (n=119)		Bronchiectasis (n=56)		COPD (n=156)		ILD (n=67)	
		N	%	N	%	N	%	N	%
Location	England	119	100.0	56	100.0	156	100.0	67	100.0
	East of England	13	10.9	9	16.1	17	10.9	9	13.4
	London	8	6.7	4	7.1	12	7.7	3	4.5
	Midlands	17	14.3	9	16.1	28	17.9	15	22.4
	North East and Yorkshire	10	8.4	4	7.1	21	13.5	7	10.4
	North West	13	10.9	5	8.9	21	13.5	9	13.4
	South East	16	13.4	9	16.1	17	10.9	6	9.0
	South West	2	1.7	2	3.6	14	9.0	6	9.0
	No answer	38	31.9	14	25.0	26	16.6	12	17.9
Other heart or lung problems	No	73	61.3	13	23.2	101	64.7	45	67.2
	Yes	20	16.8	33	58.9	46	29.5	17	25.4
	I don't know	1	0.8	1	1.8	0	0.0	0	0.0
	No answer	25	21.0	9	16.1	9	5.8	5	7.5
Vaccine (annual jab)	I don't know	13	10.9	4	7.1	3	1.9	1	1.5
	I received this vaccine	51	42.9	39	69.6	123	78.8	55	82.1
	I was not offered this vaccine	25	21.0	3	5.4	13	8.3	5	7.5
	I was offered this vaccine but did not receive it	4	3.4	1	1.8	8	5.1	1	1.5
	No answer	25	21.0	9	16.1	9	5.8	5	7.5
Vaccine (pneumonia jab)	I don't know	11	9.2	4	7.1	6	3.8	2	3.0
	I received this vaccine	22	18.5	29	51.8	93	59.6	41	61.2
	I was not offered this vaccine	57	47.9	11	19.6	35	22.4	16	23.9
	I was offered this vaccine but did not receive it	0	0.0	2	3.6	8	5.1	0	0.0
	No answer	29	24.4	10	17.9	14	9.0	8	11.9
Age	Under 18	1	0.8	0	0.0	0	0.0	0	0.0
	18–24/under40 (ILD)	3	2.5	0	0.0	1	0.6	1	1.5
	25–34/40–49 (ILD)	5	4.2	2	3.6	2	1.3	3	4.5
	35–44/50–59 (ILD)	14	11.8	1	1.8	6	3.8	17	25.4
	45–54/60–69 (ILD)	17	14.3	5	8.9	24	15.4	25	37.3
	55–64/70–79 (ILD)	22	18.5	11	19.6	58	37.2	12	17.9
	65–74/80–89 (ILD)	27	22.7	19	33.9	47	30.1	1	1.5
	75–84/90+ (ILD)	3	2.5	7	12.5	7	4.5	1	1.5
	No answer	27	22.7	11	19.6	11	7.1	7	10.4
Gender	Prefer not to say	30	25.2	11	19.6	11	7.1	8	11.9
	Female	70	58.8	37	66.1	124	79.5	39	58.2
	Male	19	16.0	8	14.3	21	13.5	20	29.9
Ethnic group	Prefer not to say	28	23.5	10	17.9	12	7.7	7	10.4
	Mixed or multiple ethnic groups	4	3.4	1	1.8	2	1.3	0	0.0
	White	85	71.4	45	80.4	142	91.0	59	88.1
	Other ethnic group	2	1.7	0	0.0	0	0.0	1	1.5
Patient advisory group	I don't belong to or follow any patient advisory groups	37	31.1	11	19.6	11	7.1	10	14.9
	Patient support group	2	1.7	7	12.5	7	4.5	16	23.9
	British Lung Foundation Breathe Easy group	13	10.9	11	19.6	32	20.5	14	20.9
	Facebook groups/pages/accounts relating to asthma	22	18.5	21	37.5	117	75.0	36	53.7
	Instagram accounts relating to asthma	3	2.5	2	3.6	5	3.2	2	3.0
	Twitter accounts relating to asthma	10	8.4	4	7.1	3	1.9	2	3.0
	Online patient forum/group	13	10.9	4	7.1	0	0.0	6	9.0
	WhatsApp group relating to asthma	1	0.8	0	0.0	0	0.0	5	7.5
	Other social media relating to asthma	5	4.2	6	10.7	8	5.1	1	1.5
	Other (please specify)	6	5.0	9	16.1	9	5.8	5	7.5
Had disease	No	11	9.2	39	69.6	53	34.0	50	74.6
	Yes	84	70.6	9	16.1	93	59.6	12	17.9
	No answer	24	20.2	8	14.3	10	6.4	5	7.5

COPD, chronic obstructive pulmonary disease; ILD, interstitial lung disease.

While only 9.2% (n=11) of respondents who were eventually diagnosed with asthma had not heard of asthma, 34% (n=53) of respondents had not heard of COPD before their diagnosis, 74.6% (n=50) had not heard of ILD and 69.6% (n=39) had not heard of bronchiectasis prior to diagnosis.

## Chronic obstructive pulmonary disease

About 76.9% (n=120) of COPD respondents self-reported that they had increased breathlessness prior to diagnosis, 45.5% (n=71) reported chest infections, 37.8% (n=59) reported they had wheezing and 37.2% (n=58) felt tiredness before seeking professional help (table 2). About 51.5% respondents noticed that their symptoms were not going away or kept recurring and 39.1% (n=61) said they were restricting daily activities. Although the individuals were aware that there was something wrong with them, the survey responses showed that more individuals did not take any actions for their symptoms 48.1% (n=75) than went to the GP (39.1%, n=61). About 58.3% (n=91) of respondents did not appreciate the severity or urgency of the situation and felt no urgency to seek professional help. About 37.2% (n=58) did not know the signs of potential COPD. Waiting time prior to a diagnosis varied across COPD respondents, from less than a week to 5–10 years (table 3). About 39.1% (n=61) of COPD respondents thought that their diagnosis had been delayed, and there were a variety of barriers to getting a diagnosis (table 4). About 19.2% (n=30) thought that it was due to the health professional’s lack of expertise or knowledge, and 18.6% (n=29) thought the healthcare professional did not take enough time to investigate their case or they reported being initially treated for another lung condition prior to a diagnosis being made (16.7%). About 10.9% respondents said that they felt they had to fight for their care.

**Table 2:** Prediagnosis experiences

Prediagnosis experiences		Asthma (n=119)		Bronchiectasis (n=56)		COPD (n=156)		ILD (n=67)	
		N	%	N	%	N	%	N	%
Self-reported symptoms before seeking professional help	Cough	75	63.0	39	69.6	75	48.1	40	59.7
	Wheezing	70	58.8	26	46.4	59	37.8	11	16.4
	Unusual phlegm/sputum	30	25.2	31	55.4	40	25.6	4	6.0
	Chest infections	48	40.3	38	67.9	71	45.5	21	31.3
	Tiredness	38	31.9	30	53.6	58	37.2	32	47.8
	Low mood	11	9.2	16	28.6	24	15.4	6	9.0
	Increased breathlessness	86	72.3	37	66.1	120	76.9	41	61.2
	Pain	11	9.2	10	17.9	15	9.6	6	9.0
	Coughing up blood	1	0.8	6	10.7	9	5.8	1	1.5
	Unusual weight changes	7	5.9	1	1.8	10	6.4	8	11.9
	Other (please specify)	12	10.1	6	10.7	13	8.3	7	10.4
Self-awareness of something was wrong	Nothing in particular, I just felt odd or generally unwell/rundown	9	7.6	3	5.4	12	7.7	4	6.0
	I was finding it more difficult to play sports or exercise	33	27.7	12	21.4	37	23.7	14	20.9
	Daily activities such as housework were more difficult to do	29	24.4	22	39.3	61	39.1	26	38.8
	Symptoms like cough or chest infections weren't going away as I would expect or they kept recurring	58	48.7	36	64.3	81	51.9	37	55.2
	It was taking me longer to recover from illness or I was not recovering fully	31	26.1	23	41.1	50	32.1	14	20.9
	A healthcare professional told me something was wrong	12	10.1	9	16.1	30	19.2	9	13.4
	Someone close to me noticed a change in me	19	16.0	6	10.7	15	9.6	8	11.9
	Other (please specify)	23	19.3	10	17.9	28	17.9	10	14.9
First action of notice something wrong	Did nothing, hoping symptoms would go away	48	40.3	15	26.8	75	48.1	15	22.4
	Used at home remedies or complementary medicine	7	5.9	8	14.3	15	9.6	5	7.5
	Searched the internet	1	0.8	1	1.8	8	5.1	3	4.5
	Spoke to family and friends	7	5.9	3	5.4	9	5.8	5	7.5
	Spoke to a pharmacist	1	0.8	2	3.6	2	1.3	0	0.0
	Sought telephone advice from NHS 111	1	0.8	1	1.8	3	1.9	0	0.0
	Went to the GP	37	31.1	23	41.1	61	39.1	31	46.3
	Felt concerned and rang 999	3	2.5	0	0.0	5	3.2	1	1.5
	Went to A&E	2	1.7	4	7.1	9	5.8	3	4.5
Motivation of seeking professional help	I was worried about my symptoms	51	42.9	29	51.8	73	46.8	37	55.2
	I was no longer able to live my life in the same way	39	32.8	26	46.4	65	41.7	26	38.8
	My partner encouraged me to seek help	11	9.2	6	10.7	25	16.0	16	23.9
	There’s a history of respiratory disease in my family so I was aware of the risks	23	19.3	9	16.1	39	25.0	8	11.9
	I was aware my job or lifestyle factors put me at risk of health issues	6	5.0	5	8.9	13	8.3	3	4.5
	I read or saw something on the internet that motivated me to take action	0	0.0	0	0.0	4	2.6	2	3.0
	I read or saw something on the TV that motivated me to take action	3	2.5	0	0.0	3	1.9	0	0.0
	I had a scare such as collapsing	9	7.6	6	10.7	16	10.3	6	9.0
	Other (please specify)	23	19.3	13	23.2	31	19.9	14	20.9
Reasons for not seeking professional help	Busy at work	14	11.8	7	12.5	40	25.6	10	14.9
	Busy with home life	12	10.1	6	10.7	30	19.2	7	10.4
	Not being registered with GP	0	0.0	0	0.0	1	0.6	0	0.0
	Not knowing the signs of potential lung disease	13	10.9	11	19.6	58	37.2	24	35.8
	Not appreciating the severity or urgency of the situation	39	32.8	16	28.6	91	58.3	38	56.7
	Not wanting to know if something was wrong	9	7.6	3	5.4	33	21.2	4	6.0
	Hoping things would go away on their own	40	33.6	15	26.8	60	38.5	19	28.4
	Concern about catching COVID-19	3	2.5	1	1.8	1	0.6	3	4.5
	Not being able to get an appointment at a time that suited me	5	4.2	1	1.8	4	2.6	5	7.5
	Other (please specify)	13	10.9	11	19.6	18	11.5	14	20.9
Symptoms worsen between first seeking professional help and getting diagnosis	I don't know	19	16.0	9	16.1	20	12.8	11	16.4
	No	25	21.0	9	16.1	54	34.6	13	19.4
	Yes	51	42.9	30	53.6	73	46.8	38	56.7
	No answer	24	20.2	8	14.3	9	6.0	5	7.5
Living time with symptoms	Less than 1 month	18	15.1	4	7.1	10	6.4	8	11.9
	1–2 months	18	15.1	4	7.1	10	6.4	12	17.9
	3–6 months	16	13.4	3	5.4	26	16.7	13	19.4
	7–12 months	6	5.0	7	12.5	27	17.3	11	16.4
	1–2 years	7	5.9	3	5.4	35	22.4	11	16.4
	3–5 years	7	5.9	8	14.3	20	12.8	6	9.0
	10 years	5	4.2	0	0.0	5	3.2	0	0.0
	10+ years	6	5.0	9	16.1	5	3.2	1	1.5
	I don't remember	15	12.6	11	19.6	12	7.7	1	1.5
	No answer	21	17.6	7	12.5	6	3.8	4	6.0

COPD, chronic obstructive pulmonary disease; ILD, interstitial lung disease; NHS, National Health Service.

**Table 3:** Diagnosis pathway

Diagnosis pathway		Asthma (n=119)		Bronchiectasis (n=56)		COPD (n=156)		ILD (n=67)	
		N	%	N	%	N	%	N	%
Waiting time	Less than 1 week	20	16.8	2	3.6	20	12.8	3	4.5
	1–2 weeks	5	4.2	0	0.0	0	0.0	0	0.0
	2–4 weeks	0	0.0	3	5.4	19	12.2	2	3.0
	1–2 months	4	3.4	4	7.1	16	10.3	10	14.9
	3–6 months	8	6.7	4	7.1	16	10.3	18	26.9
	7–12 months	6	5.0	6	10.7	16	10.3	12	17.9
	1–2 years	5	4.2	8	14.3	11	7.1	11	16.4
	5 years	9	7.6	8	14.3	13	8.3	5	7.5
	10 years	6	5.0	1	1.8	2	1.3	1	1.5
	10+ years	4	3.4	4	7.1	4	2.6	1	1.5
	I don't remember	19	16.0	7	12.5	15	9.6	4	6.0
	No answer	0	0.0	8	14.3	8	5.1	0	0.0
First diagnosis time	Before 2000	34	28.6	8	14.3	8	5.1	2	3.0
	2000–2009	18	15.1	4	7.1	26	16.7	39	58.2
	2010–2019	26	21.8	21	37.5	76	48.7	19	28.4
	2020 Onward	9	7.6	9	16.1	14	9.0	6	9.0
	No answer	32	26.9	14	25.0	32	20.5	1	1.5
Diagnosis Location	A&E	3	2.5	0	0.0	4	2.6	2	3.0
	GP practice	54	45.4	2	3.6	82	52.6	4	6.0
	At home	4	3.4	4	7.1	6	3.8	7	10.4
	Hospital outpatients department (other clinic)	1	0.8	2	3.6	2	1.3	4	6.0
	Hospital outpatients department (respiratory clinic)	17	14.3	27	48.2	30	19.2	34	50.7
	None of the above/I don't know	4	3.4	8	14.3	1	0.6	5	7.5
	Other (please specify)	6	5.0	7	12.5	11	7.1	3	4.5
	While admitted as a hospital inpatient	7	5.9	6	10.7	12	7.7	8	11.9
Diagnosis process/test	Discussion with my doctor	66	55.5	17	30.4	50	32.1	21	31.3
	Spirometry (breathing test where you blow hard into a mouthpiece on a small machine)	43	36.1	21	37.5	108	69.2	33	49.3
	6-Minute walking test	4	3.4	4	7.1	17	10.9	16	23.9
	Chest X-ray	25	21.0	26	46.4	73	46.8	35	52.2
	Oxygen saturation (finger probe) test	13	10.9	15	26.8	30	19.2	20	29.9
	Phlegm (sputum) test	9	7.6	12	21.4	15	9.6	2	3.0
	Sweat test	0	0.0	3	5.4	0	0.0	0	0.0
	CT scan	14	11.8	33	58.9	34	21.8	46	68.7
	Other scan	0	0.0	3	5.4	2	1.3	3	4.5
	Bronchoscopy (when a flexible tube is put into your nose or mouth and into your lungs)	2	1.7	8	14.3	5	3.2	9	13.4
	Lung biopsy	1	0.8	0	0.0	1	0.6	10	14.9
	Bronchoalveolar lavage (when liquid is put through the bronchoscope to get a sample)	0	0.0	3	5.4	2	1.3	3	4.5
	Blood tests	13	10.9	15	26.8	34	21.8	25	37.3
	Feno testing (test where you breath out slowly through a filter into a portable machine)	10	8.4	5	8.9	9	5.8	2	3.0
	Pulmonary exercise stress test	2	1.7	1	1.8	10	6.4	2	3.0
	Histamine testing (skin prick test to look for allergies)	10	8.4	3	5.4	3	1.9	2	3.0
	Other (please specify)	13	10.9	7	12.5	3	1.9	7	10.4
Risk factors	Smoking	14	11.8	8	14.3	122	78.2	14	20.9
	Passive smoking	21	17.6	7	12.5	34	21.8	4	6.0
	Genetic factors	43	36.1	11	19.6	46	29.5	11	16.4
	Poor housing (eg, damp, mould, living near sewage)	17	14.3	4	7.1	15	9.6	7	10.4
	Air pollution	26	21.8	6	10.7	33	21.2	12	17.9
	Exposure at work	12	10.1	2	3.6	29	18.6	16	23.9
	Previous infections	25	21.0	21	37.5	32	20.5	10	14.9
	Poor health	11	9.2	7	12.5	11	7.1	4	6.0
	Other (please specify)	17	14.3	20	35.7	19	12.2	14	20.9

COPD, chronic obstructive pulmonary disease; ILD, interstitial lung disease.

**Table 4:** Diagnosis delay issues

Diagnosis delay issues		Asthma (n=119)		Bronchiectasis (n=56)		COPD (n=156)		ILD (n=67)	
		N	%	N	%	N	%	N	%
Feel diagnosis has been delayed	I don't know	18	15.1	8	14.3	16	10.3	4	6.0
	No	36	30.3	12	21.4	70	44.9	23	34.3
	Yes	41	34.5	32	57.1	61	39.1	35	52.2
	No answer	24	20.2	4	7.3	9	5.7	5	7.5
Effect of diagnosis delay	I worried for longer than I needed to	13	10.9	14	25.0	24	15.4	20	29.9
	I felt my condition worsened more than it had to	33	27.7	21	37.5	30	19.2	16	23.9
	I became demotivated	6	5.0	6	10.7	11	7.1	5	7.5
	My mental health suffered	14	11.8	12	21.4	24	15.4	20	29.9
	I felt like I wasn't being taken seriously	21	17.6	21	37.5	34	21.8	16	23.9
	I didn’t get medication quickly enough	29	24.4	25	44.6	30	19.2	16	23.9
	I didn’t get advice on managing my condition quickly enough	28	23.5	25	44.6	42	26.9	24	35.8
	Other (please specify)	11	9.2	10	17.9	10	7.4	7	10.4
Barriers to getting a diagnosis	I do not recall there being any barriers	32	26.9	5	8.9	45	28.8	17	25.4
	Being treated for another lung condition	10	8.4	25	44.6	26	16.7	12	17.9
	Being treated for another non-lung condition (eg, heart condition)	3	2.5	1	1.8	9	5.8	6	9.0
	Symptoms were attributed to a pre-existing condition that I had	12	10.1	19	33.9	16	10.3	7	10.4
	Lack of expertise or knowledge, eg, Healthcare professional not recognising my symptoms	29	24.4	19	33.9	30	19.2	20	29.9
	Lack of effort or motivation, eg, Healthcare professional not taking the time to investigate	29	24.4	15	26.8	29	18.6	12	17.9
	Feeling like I had to fight for my care, eg, being turned away by my GP	21	17.6	15	26.8	17	10.9	15	22.4
	Difficulty getting appointments	12	10.1	8	14.3	24	15.4	9	13.4
	Long waiting times or delays	6	5.0	10	17.9	20	12.8	15	22.4
	Lack of follow-up to discuss test results	8	6.7	9	16.1	25	16.0	9	13.4
	COVID-19	2	1.7	2	3.6	5	3.2	8	11.9
	I was misdiagnosed	13	10.9	6	10.7	16	10.3	14	20.9
	Other (please specify)	9	7.6	9	16.1	14	9.0	8	11.9

COPD, chronic obstructive pulmonary disease; ILD, interstitial lung disease.

### Asthma

Respondents self-reported that the most common prediagnosis symptoms were increased breathlessness (72.3%, n=86), cough (63.0%, n=75), wheezing (58.8%, n=70), chest infections (40.3%, n=48) and unusual phlegm/sputum (25.2%, n=30) (table 2). About 27.7% (n=33) of respondents complained about difficulty playing sports or exercising, and 24.4% (n=29) claimed that their daily activities such as housework were more difficult. About 32.8% (n=39) of respondents did not appreciate the severity of their presymptoms and felt no urgency to seek professional help. Although the majority of respondents were aware that there was something wrong with them, 40.3% (n=48) of asthma patients were not taking any action for their symptoms, and only 31.1% (n=37) individuals went to the GP to get professional help. The diagnostic waiting time varied for people with asthma, from less than a week to more than 10 years, but the most common response was less than 1 week (16.8%, n=20); overall, most people with asthma received a diagnosis within 6 months (table 3). About 34.5% (n=41) of respondents thought that their diagnosis had been delayed (table 4). The delayed diagnoses led 27.7% (n=33) of respondents to feel that their condition worsened unnecessarily and 17.6% (n=21) of patients to feel they were not being taken seriously. About 24.4% (n=29) said they did not get medication quickly enough, and almost same number (n=28) thought they did not get advice on managing their condition quickly enough either.

### Interstitial lung disease

The most common prediagnostic symptoms were increased breathlessness (61.2%, n=41), cough (59.7%, n=40) and tiredness 47.8% (n=32) (table 2). About 55.2% (n=37) respondents noticed that their symptoms were not going away or kept recurring. About 38.8% (n=26) claimed their daily activities such as housework were more difficult to do. However, 13.4% of respondents relied on the healthcare professionals to tell them that something was wrong or someone close to them noticed a change in them (11.9%). Less than half (n=31, 46.3%) went to the GP to get professional help. About 53.7% (n=38) claimed that their symptoms worsened between first seeking professional help and getting a diagnosis. About 56.7% (n=38) of respondents did not appreciate the severity or urgency of the situation, and 35.8% (n=24) did not know the signs of potential ILD disease. The diagnosis waiting time varied with patients with ILD, from less than a week to more than 10 years, but the most common response was 3–6 years (n=18, 26.9%) (table 3). More than half (50.7%, n=34) reported that they were diagnosed at a hospital outpatients department (respiratory clinic), only 6.0% (n=4) of respondents were diagnosed at GP practice, and 11.9% (n=8) said they were diagnosed while they were admitted as a hospital inpatient. About 52.2% (n=35) of respondents thought that their diagnosis had been delayed (table 4), and 35.8% (n=24) respondents reported that they did not get advice on managing their condition quickly enough. About 20.9% (n=14) of respondents reported that they were misdiagnosed.

### Bronchiectasis

The most common prediagnosis symptoms reported were cough (69.6%, n=39), chest infection (67.9%, n=38), increased breathlessness (66.1%, n=37) and unusual phlegm/sputum (55.4%, n=31) (table 2). Although the individuals were aware that there was something wrong with them, 26.8% (n=15) patients were not taking any actions for their symptoms. Then only 41.1% (n=23) individuals went to the GP to get professional help. About 51.8% (n=29) respondents claimed that they were worried about their symptoms, which motivated them to seek professional help. About 46.4% (n=26) respondents sought professional help because they felt that they were no longer able to live their life in the same way. The diagnosis waiting time varied, from less than a week to 5–10 years, but the most common responses were 1–2 years (14.3%) and 2–5 years (14.3%) (table 3). About 57.1% (n=32) of patients with bronchiectasis thought that their diagnosis had been delayed (table 4). Almost half (44.6%) the respondents claimed they were being initially treated for another lung condition. About 33.9% (n=19) reported the delayed diagnosis was due to the health professionals’ lack of expertise or knowledge, and a similar number claimed that health professionals were not aware of the symptoms’ difference from a pre-existing condition that the patients had. The delayed diagnoses led 37.5% (n=21) respondents feel like that they were not being taken seriously by their health professionals, and the same proportion felt their condition worsened as a result.

### Comparing across the diseases

While there were a number of similarities, there were some differences across the four diseases of patients’ prediagnostic experiences. First, people with COPD were more likely (37.2%) and people with asthma less likely (10.9%) to report they did not know the signs of potential lung disease (p<0.001) (table 5). People with COPD were more likely to report that they did not appreciate the severity or urgency of the situation (58.3%) than people with asthma (32.8%), ILD (43.3%) and bronchiectasis (28.6%, p<0.001). Patients with COPD were statistically significantly more likely (p<0.001) to not want to know if something was wrong (21.2%) than the other groups (asthma: 7.6%, ILD: 6.0% and bronchiectasis: 5.4%).

**Table 5:** Comparing across the diseases

Statistical differences across the four diseases	Asthma (n=119)		Bronchiectasis (n=56)		COPD (n=156)		ILD (n=67)		P value
	N	%	N	%	N	%	N	%	
Not knowing the signs of potential lung disease	13	10.9	11	19.6	58	37.2	24	35.8	<0.001
Not appreciating the severity or urgency of the situation	39	32.8	16	28.6	91	58.3	38	56.7	<0.001
Not wanting to know if something was wrong	9	7.6	3	5.4	33	21.2	4	6.0	<0.001
I worried for longer than I needed to	13	10.9	14	25.0	24	15.4	20	29.9	<0.001
Long waiting times or delays	6	5.0	10	17.9	20	12.8	15	22.4	<0.001
Being treated for another lung condition	10	8.4	25	44.6	26	16.7	12	17.9	<0.001
Spirometry test	43	36.1	21	37.5	108	69.2	33	49.3	<0.001
Chest X-ray	25	21.0	26	46.4	73	46.8	35	52.2	<0.001
Oxygen saturation test	13	10.9	15	26.8	30	19.2	20	29.9	<0.001
Blood tests	13	10.9	15	26.8	34	21.8	25	37.3	<0.001

COPD, chronic obstructive pulmonary disease; ILD, interstitial lung disease.

Second, during the diagnostic process, people with COPD were more likely to have spirometry (69.3%) and people with asthma less likely (36.1%) than expected under the null hypothesis of no difference between the groups (p <0.001). People with asthma (21.0%, p<0.001) were less likely than people with ILD (52.2%), COPD (46.8%) or bronchiectasis (46.4%) to have a chest X-ray. Oxygen saturation test also differed significantly by group (p =0.007), as people with asthma were less likely to report this (10.9%) and people with ILD more likely to report this (29.9%) than expected. Blood tests (p<0.001) were less often reported by patients with asthma (10.9%) and more often reported by patients with ILD (37.3%) than expected.

Finally, for diagnostic delay, the proportions of patients reporting that they were being treated by their heath professional for another lung condition differed statistically (p<0.001) across the groups: this was more likely in people with bronchiectasis (44.6%) and less likely in people with asthma (8.4%) than expected. Patients with ILD worried most for longer than they needed to get the diagnosis (p=0.004) at 29.2% compared with patients with COPD (15.4%), asthma (10.9%) and bronchiectasis (10.9%). Patients with ILD also were the likeliest to report a long waiting time (p=0.004) at 22.4% compared with COPD (12.8%), asthma (5.0%), and bronchiectasis (17.9%).

Using χ^2^ tests, we also examined the association between those responders who had standard testing for their lung condition including spirometry for COPD and asthma, CT scan for ILD, CT and sputum collection for bronchiectasis and their perceptions of barriers and delay in receiving a diagnosis. There was no statistical evidence for a difference for any disease (table 6).

**Table 6:** Association between testing and the responders’ perceptions of barriers and delay in receiving a diagnosis

	Perceived barrier			Perceived delay		
	Yes	Total	P value	Yes	Total*	P value
COPD (spirometry)						
No	37 (77.1%)	48		20 (55.6)	36	
Yes	74 (68.5%)	108	0.276	41 (43.2)	95	0.204
Asthma (spirometry)						
No	56 (73.7)	76		20 (48.8)	41	
Yes	31 (72.1)	43	0.851	21 (58.3)	36	0.402
ILD (CT)						
No	17 (81.0)	21		9 (64.3)	14	
Yes	33 (71.7)	46	0.421	26 (59.1)	44	0.729
Bronchiectasis (CT or sputum)						
No	22 (95.7)	23		17 (77.3)	22	
Yes	29 (87.9)	33	0.502	15 (68.2)	22	0.498

*For perceived delay, we excluded don't know and missing data (25 patients with COPD, 42 patients with asthma, 12 patients with bronchiectasis and 9 patients with ILD).

COPD, chronic obstructive pulmonary disease; ILD, interstitial lung disease.

### Free test responses from the respondents

Seven questions (online supplemental appendix 3) were analysed using Braun and Clarke’s six-phase approach to thematic analysis.^9^ In total, 641 codes were coded (176 codes for COPD, 171 codes for asthma, 123 codes for ILD and 171 codes for bronchiectasis). Breathing problems were the most common symptoms perceived by all of the respondents. Response to risk factors varied between these four diseases; some risk factors were modifiable (such as pollution or work exposure) while some were non-modifiable factors (such as genetic). ‘Struggled with walking and breathing’ was the most common reason that caused the respondents to notice something was wrong and motivated them to seek medical help.

Free text responses also highlighted different reasons/barriers for delayed diagnosis and its effects on the patients. We categorised these into two main themes and four subthemes—Theme 1: personal role in diagnosis (patients lack knowledge and awareness of presymptoms, personal behaviours delayed diagnosis; Theme 2: healthcare professional’s role in diagnosis (healthcare professionals lack knowledge and expertise, healthcare professionals’ attitudes affect diagnosis).

### Theme 1: personal role in diagnosis

#### Patients’ lack of knowledge and awareness of prediagnosis symptoms

From the self-reported survey, it can be seen that a part of the reason for delayed diagnosis is due to the patients’ lack of knowledge and awareness of prediagnosis symptoms. For example, one COPD respondent said ‘Hoping breathlessness would decrease after stopping smoking. Also thought it might be exercise induced breathlessness due to heart condition’. Another patient with ILD said: ‘It’s just a cough! Thought it was ageing, slowing down’. Similarly, two other respondents said ‘I didn’t know any different, I thought it is what it is’ and ‘Ignorance as I'd never heard of bronchiectasis’. A lack of knowledge and being unaware of symptoms prior to diagnosis from the personal side would have caused diagnosis delay in some patients.

### Personal behaviours delayed diagnosis

Patients’ behaviours are also playing a role in diagnosis delay. For example, one patient with COPD said: ‘I delayed my own diagnosis by burying my head in the sand’. Trying to avoid thinking about their illness led them to seek medical help late. One person with asthma said: ‘I didn't want to find out what I felt I already suspected.’ A respondent with COPD gave the identical quote. Therefore, personal behaviours of not seeking timely medical support and unwillingness to confront their illness or avoiding their poor health condition are also reasons for delayed diagnosis.

### Theme 2: healthcare professionals’ role in diagnosis

The respondents also claimed that health professionals played an important role in the delayed diagnosis. Some respondents reported that the delayed diagnosis was due to their healthcare professionals’ lack of knowledge and expertise, while others think that their healthcare professionals’ attitudes affect their diagnosis and caused stress for them. For example, one respondent said: ‘Dr XXX the original doctor I used under a private health insurance policy was completely wrong. I took a further 9 years to fully establish my condition, but once it was, I was escalated up the chain of care quickly.’

### Healthcare professionals’ lack knowledge and expertise

A few respondents claimed that healthcare professionals were unable to recognise the early signs or lacked knowledge of the diseases, which may have led to a delayed diagnosis. For example, one patient with COPD said: ‘GP originally said that everyone had a cough and it would go away in summer. Took three severe chest infections before I was sent to the hospital by a new young doctor under the two week rule’. A respondent with bronchiectasis reported: ‘I was treated three times for chest infections. Two months later they were back each time. Finally sent for X-ray. No face to face appointment and told over the phone. No referral no advice’. Another respondent reported a similar situation:

I had a chest x-ray that showed areas of hyperinflation. I was told that was normal and nothing more was said for another 2 years. I had an exacerbation and the doctor gave me antibiotics and a blue salbutamol inhaler. I thought the exacerbation was made worse by using the blue inhaler.

Similarly, another patient said: ‘Felt my GP didn’t have a clue, and kept telling me it was hay fever. Ended up crying my eyes out in the surgery because I couldn’t breathe’.

Misdiagnosis was also reported as one of the reasons for the delayed diagnosis. For example, a number of respondents reported that: ‘I was diagnosed with a chest infection first’; ‘Doctors kept telling me it was asthma’; ‘was initially diagnosed with asthma’; ‘was treated for asthma which i don’t have’.

Due to a delayed diagnosis, one respondent complained that: ‘If the original infection had been identified when I first visited the GP the damage to my lungs may not have been so bad.’ So from the above free text responses, it can be seen that healthcare professionals’ expertise in recognising presymptoms and detecting early signs of the disease delayed the diagnosis in some patients.

### Healthcare professionals’ attitudes affect the diagnosis

The survey report shows that healthcare professionals’ attitudes of not taking the patient’s condition seriously prolonged the diagnosis process, as participants reported: ‘Not being taken seriously by GP’ and ‘Delayed referral GP not believing my symptoms had changed’. One respondent was challenged by multiple GP visits until the final diagnosis: ‘I didn’t get a referral to respiratory medicine until after many requests, I visited my GP 22 times in one year they told me it’s anxiety’. This is consistent with another participant who said: ‘Dr only did spirometer test years after I had first seen him for persistent cough and chest infections’.

Appointment unavailability also caused the delayed diagnosis for some people: ‘I was treated three times for chest infections. Two months later they were back each time. Finally sent for X-ray. No face to face appointment and told over the phone. No referral no advice’. ‘Appointments cancelled more than once’.

Regarding the effects of delayed diagnosis, some respondents reported that it made their physical health worse and that their disease remained uncontrolled. They also said it created mental distress for them. For example, one respondent reported: ‘I felt as if no one in the medical profession cared about me’; one respondent with asthma also said the same thing: ‘I felt unimportant and ignored’.

Even after diagnosis, the respondent continually perceived the experience of being uncared for as reported: ‘I continue to experience difficulties with seeking timely help even since diagnosis. Lots of HCPs (A&E, GPs, practice nurses, etc) are not aware of bronchiectasis as a condition, exacerbations etc and how to manage’.

The free text responses were largely consistent with the rest of the survey.

## Discussion

This survey exposed many issues on the route to diagnosis of four important respiratory diseases. Patients do not appear to be satisfied in general with the current diagnostic process as evidenced by the time it takes to receive a diagnosis, the investigation pathway to that diagnosis and perhaps most importantly, a feeling of lack of healthcare professionals’ awareness leading to delay in diagnosing the disease. The survey also disclosed patients' lack of personal knowledge and awareness of symptoms, as many had not heard of the diseases before their diagnosis, did not recognise the symptoms of their diseases and did not appreciate their severity. Therefore, patients did not always take action or seek support for their symptoms. Across the survey, almost half of respondents said they were hoping symptoms would go away by themselves, so better symptom recognition and awareness may promote more timely diagnosis and better healthcare outcomes.

Survey responses also showed that the delayed diagnosis was related to healthcare providers. Reasons included healthcare professionals' lack of expertise, not taking enough time to investigate the issue, and difficulty in getting an appointment. Diagnostic errors and the issue of level of knowledge of respiratory diseases of healthcare professionals have also been reported in previous studies.^10–12^ The perception of a lack of discussion may reflect a failure in the diagnostic process too. Consistent evidence shows that insufficient communication is associated with negative health outcomes.^13 14^ To better understand the reasons behind the misdiagnosis, the delayed diagnosis and the current state of the diagnostic process with respiratory diseases in the NHS, further studies with appropriate research methodology for a more in-depth study should be conducted.

Other indicators such as location of diagnosis, diagnosis waiting time and the most common symptoms across these four diseases deserve mention. For example, most patients with asthma and COPD were diagnosed at a GP practice, whereas bronchiectasis and ILD were diagnosed mostly at hospital outpatients. Furthermore, the survey responses show that less than half of patients with ILD, asthma and bronchiectasis received the spirometry test during their diagnosis even though it is a common investigation on the diagnostic pathway. In contrast, two out of three patients with COPD had a spirometry test. The absence of standard testing could lead to delayed diagnosis or mislabelling of respiratory disease and is likely to have been compounded by the COVID-19 pandemic. However, there was no strong statistical evidence for this relation in our study, though this may be due to its limited sample sizes. Previous studies have demonstrated the role of spirometry in the diagnosis of respiratory disease and showed a significant effect on the outcome of timely diagnosis.^15–17^ One longitudinal study with patients with COPD also evidenced that diagnosis using pulmonary function testing reduces mortality and morbidity rates and decreases admission to hospital.^7^ Why there is such a low rate of patients receiving spirometry, and what the effect is of a lack of pulmonary function screening on patients, needs to be studied further. Other diagnostic tests, such as blood tests, phlegm test and bronchoscopy, also showed statistically significant differences across the groups.

Improving diagnostic services not only requires health professionals to understand and use a personalised approach for the diagnosis process, but it also needs the patients to improve their knowledge of symptom severities and assessment management. The survey suggests that part of the failure is on the part of clinicians to educate patients about their diagnosis. Many respiratory symptoms and diseases are more likely to develop into a long-term condition, and much can be done to prevent disease and minimise further deterioration once it has begun. Patients recognising symptoms and seeking early help are the first steps to getting a timely diagnosis, while clinicians’ knowledge and response are key to achieve a person-centred diagnosis. This study is the first survey across the four most common respiratory diseases of patient experiences and perceptions regarding the diagnostic process in England. It highlights certain difficulties in the diagnostic process for long-term respiratory diseases on the part of both the patient and healthcare provider.

## Limitations

The dataset is relatively small, and a larger sample size would be needed to further assess the effect of a timely diagnosis on respiratory-related healthcare service utilisation. This is a cross-sectional questionnaire, and results are potentially subject to non-response bias and recall bias. A solution to reducing the latter would be to verify the respondents’ results with their medical records, but this was not possible due to respondents’ anonymity and would in any case not pick up patients’ opinions and knowledge. As this study was advertised primarily via the charity, selection bias may occur as the respondents are in an active online group. Furthermore, all the respondents were resident in England, and experience may differ in the rest of the UK or in other countries. Similarly, the participants were predominantly white, who may have different experiences of using healthcare services from other groups. Lastly, the survey did not address comorbidity.

## Conclusions

The study identified some differences of perceived experiences of patients with respiratory diseases. We found that the diagnostic process was perceived as being prolonged, and reasons for delayed diagnosis were mainly due to healthcare professionals and patients lacking the knowledge to recognise symptoms. These findings indicate the importance of promoting patients’ knowledge of their diseases. It is important to improve awareness of the signs and symptoms of chronic respiratory diseases for people and to empower healthcare professionals to recognise the barriers that contribute to diagnosis delay and to facilitate earlier diagnosis and management.

## Data Availability

All data relevant to the study which can be shared are included in the article or uploaded as supplementary information.
