# Supplementary material for: Survey-identified experiences of prediagnosis and diagnosis process among patients with COPD, asthma, interstitial lung disease and bronchiectasis
Source: BMJ Open Respir Res. 2023 Nov 22;10(1):e001588. doi: 10.1136/bmjresp-2022-001588 (PMC10668245; doi:10.1136/bmjresp-2022-001588)
Supplement: Supplementary data [file bmjresp-2022-001588supp001.pdf]

Appendix 1 Survey Questionnaires

1. Have you been diagnosed with asthma?

Someone with 'severe asthma' has a specific type of asthma which doesn't get better with the usual medicines. Even if someone takes those medicines exactly as prescribed, a different approach is needed to control symptoms and reduce frequent asthma attacks. Severe asthma is usually diagnosed in a specialist severe asthma hospital.

○ Yes, I have been diagnosed with asthma

○ Yes, I have been diagnosed with severe asthma

○ No, I have not been diagnosed with asthma

2. Which country in the UK do you currently live in?

○ England

○ Scotland

○ Wales

○ Northern Ireland

○ I don't live in the UK

3. Thinking just about your diagnosis of asthma, which of the following symptoms did you experience before seeking professional help (e.g. visiting a GP or A&E)? Please tick all that apply

○ Increased breathlessness

○ Cough

○ Wheezing

○ Unusual phlegm / sputum

○ Chest infections

○ Tiredness

○ Low mood

○ Pain

○ Coughing up blood

○ Unusual weight changes

○ Other (please specify)

○ None of the above / I don't know

4. What caused you to notice something was wrong? Please tick all that apply

○ Nothing in particular, I just felt odd or generally unwell/rundown

○ I was finding it more difficult to play sports or exercise

○ Daily activities such as housework were more difficult to do

○ Symptoms like cough or chest infections weren't going away as I would expect or they kept recurring

○ It was taking me longer to recover from illness or I was not recovering fully

○ A healthcare professional told me something was wrong

○ Someone close to me noticed a change in me

○ Other (please specify)

○ None of the above / I don't know

5. What did you do after you noticed you were experiencing symptoms?

This question is asking about what you did when you first noticed your symptoms, up to and including when you first sought professional help (e.g. visiting a GP or A&E). So does not include referrals or specialist appointments that may have happened later on.

Please rank these in the order that you did them. It may be best to read through all the options before answering. For options that don't apply to you, select 'N/A'.

If you did multiple things at once, please give them the same rank. If you can't remember the order, please try to pick at least one thing you did first and order all the other things you did equally. (e.g. all as 2nd or all as 3rd)

	1st	2nd	3rd	4th	5th	6th	7th	8th	N/A
Did nothing, hoping symptoms would go away	○	○	○	○	○	○	○	○	○
Used at home remedies or alternative medicine	○	○	○	○	○	○	○	○	○
Searched the internet	○	○	○	○	○	○	○	○	○
Spoke to family & friends	○	○	○	○	○	○	○	○	○
Spoke to a pharmacist	○	○	○	○	○	○	○	○	○
Sought telephone advice from NHS 111	○	○	○	○	○	○	○	○	○
Went to the GP	○	○	○	○	○	○	○	○	○
Felt concerned and rang 999	○	○	○	○	○	○	○	○	○
Went to A&E	○	○	○	○	○	○	○	○	○

6. How long did you live with your asthma symptoms before seeking professional help (e.g. visiting a GP or A&E)?

Common symptoms include increasing breathlessness, a persistent chesty cough with phlegm that does not go away, frequent chest infections and persistent wheezing.

○ Less than 1 month

○ 1 - 2 months

○ 3 - 6 months

○ 7 - 12 months

○ 1 - 2 years

○ 3 - 5 years

- 6- 10 years
- 10 + years
- I don't know / not applicable

7. What motivated you to seek professional help? Please tick all that apply

- I was worried about my symptoms
- I was no longer able to live my life in the same way
- My partner encouraged me to seek help
- There's a history of respiratory disease in my family so I was aware of the risks
- I was aware my job or lifestyle factors put me at risk of health issues
- I read or saw something on the internet that motivated me to take action I read or saw something on the TV that motivated me to take action
- I had a scare such as collapsing
- Other (please specify)
- None of the above / I don't know

8. What, if anything, prevented you from seeking professional help earlier? Please tick all that apply

- Busy at work
- Busy with home life
- Not being registered with a GP
- Not knowing the signs of potential lung disease
- Not appreciating the severity or urgency of the situation
- Not wanting to know if something was wrong
- Hoping things would go away on their own
- Concern about catching Covid
- Not being able to get an appointment at a time that suited me
- Other (please specify)
- None of the above / I don't know

9. How long did you have to wait between seeking / receiving professional help for your asthma (e.g. visiting a GP or A&E) and receiving a formal diagnosis? Please estimate if you're not sure

- Less than 1 week
- 1 - 2 weeks
- 2 - 4 weeks
- 1 - 2 months
- 3 - 6 month
- 7 - 12 months
- 1 - 2 years
- 2 - 5 years
- 5- 10 years
- 10+ years
- I don't remember

10. What year were you first diagnosed with asthma? Please give your best guess if you can't remember.

11. Where were you when you were given your asthma diagnosis?

- ○ GP practice
- ○ A&E
- ○ Whilst admitted as a hospital inpatient
- ○ Hospital outpatients department (respiratory clinic)
- ○ Hospital outpatients department (other clinic)
- ○ At home (told of my diagnosis over the phone)
- ○ Other (please specify)
- ○ None of the above / I don't know

12. How were you diagnosed? Please tick all that apply

- ○ Discussion with my doctor
- ○ Spirometry (breathing test where you blow hard into a mouthpiece on a small machine)
- ○ 6-minute walking test
- ○ Chest x-ray
- ○ Oxygen saturation (finger probe) test
- ○ Phlegm (sputum) test
- ○ Sweat test
- ○ CT scan
- ○ Other scan
- ○ Bronchoscopy (when a flexible tube is put into your nose or mouth and into your lungs)
- ○ Lung biopsy
- ○ Bronchoalveolar lavage (when liquid is put through the bronchoscope to get a sample)
- ○ Blood tests
- ○ Feno testing (test where you breath out slowly through a filter into a portable machine)
- ○ Pulmonary Exercise Stress Test
- ○ Histamine testing (skin prick test to look for allergies)
- ○ Other (please specify)
- ○ None of the above / I don't know

13. Had you heard of asthma before you were diagnosed?

- ○ Yes
- ○ No
- ○ I don't know

14. Did your symptoms worsen between first seeking professional help and getting your diagnosis?

- ○ Yes
- ○ No
- ○ I don't know

15. Do you feel your diagnosis was delayed?

- ○ Yes
- ○ No
- ○ I don't know

16. If you feel that your diagnosis was delayed, how did this affect you? Please tick all that apply

- ○ I worried for longer than I needed to

- I felt my condition worsened more than it had to
- I became demotivated
- My mental health suffered
- I felt like I wasn't being taken seriously
- I didn’t get medication quickly enough
- I didn’t get advice on managing my condition quickly enough
- Other (please specify)
- None of the above / I don't know

17. What were the main barriers, if any, to getting a diagnosis? Please tick all that apply

- Being treated for another lung condition
- Being treated for another non-lung condition (e.g. heart condition)
- Symptoms were attributed to a pre-existing condition that I had
- Lack of expertise or knowledge, e.g. Healthcare professional not recognising my symptoms
- Lack of effort or motivation, e.g. Healthcare professional not taking the time to investigate
- Feeling like I had to fight for my care, e.g. being turned away by my GP
- Difficulty getting appointments
- Long waiting times or delays
- Lack of follow up to discuss test results
- COVID-19
- I was misdiagnosed
- Other (please specify)
- I do not recall there being any barriers

18. What factors do you think contributed to your asthma? Please tick all that apply

- Smoking
- Passive smoking
- Genetic factors
- Poor housing (e.g. damp, mould, living near sewage)
- Air pollution
- Exposure at work
- Previous infections
- Poor health
- Other (please specify)
- None of the above / I don't know

19. Had you already been diagnosed with any heart or lung problems prior to your asthma diagnosis?

- Yes
- No
- I don't know

If yes, please specify the name of these

20. In the year after being diagnosed, were you offered the following vaccines?

	I received this vaccine	I was offered this vaccine but did not receive it	I was not offered this vaccine	I don't know
Annual flu jab	○	○	○	○
Pneumonia Jab	○	○	○	○

Questions about you help us understand if healthcare is being delivered fairly to all people no matter their location, age, gender, ethnicity or socio-economic background.

19. What was your postcode leading up to your diagnosis? Please provide your full postcode.

By survey participants providing their postcodes it allows us to explore how the delivery of healthcare varies across country, between local areas and between different groups of people. Using this we can understand if healthcare is being delivered fairly to all people or if certain socio- economic factors affect your care.

20. What is your age?

○ Under 18

○ 18-24

○ 25-34

○ 35-44

○ 45-54

○ 55-64

○ 65-74

○ 75-84

○ 85-94

○ 95+

○ Prefer not to say

21. What is your gender?

○ Male

○ Female

○ I prefer to self describe

○ I would prefer not to say

22. Which of these best describes your ethnic group?

○ White

○ Mixed or Multiple ethnic groups

○ Asian or Asian British

○ Black, African, Caribbean or Black British

○ Arab

○ Prefer not to say

○ Other ethnic group (please specify)

23. Do you belong to or follow any patient advisory groups? Please tick all that apply

- ○ Patient support group
- ○ British Lung Foundation Breathe Easy group
- ○ Facebook groups/pages/accounts relating to asthma
- ○ Instagram accounts relating to asthma
- ○ Twitter accounts relating to asthma
- ○ Other social media relating to asthma
- ○ Online patient forum/group
- ○ WhatsApp group relating to asthma
- ○ Other (please specify)
- ○ I don't belong to or follow any patient advisory groups

Appendix 2 Examples of Chi-squared test

disease * Not appreciating the severity or urgency of the situation

Crosstab

			Not appreciating the severity or urgency of the situation		
				Not appreciating the severity or urgency of the situation	Total
disease	Asthma	Count	80	39	119
		% within disease	67.2%	32.8%	100.0%
		Standardized Residual	1.6	-1.8	
	COPD	Count	65	91	156
		% within disease	41.7%	58.3%	100.0%
		Standardized Residual	-2.4	2.7	
	Broch	Count	40	16	56
		% within disease	71.4%	28.6%	100.0%
		Standardized Residual	1.5	-1.7	
	ILD	Count	38	29	67
		% within disease	56.7%	43.3%	100.0%
		Standardized Residual	.1	-.1	
Total		Count	223	175	398
		% within disease	56.0%	44.0%	100.0%

Chi-Square Tests

	Value	df	Asymptotic Significance (2-sided)
Pearson Chi-Square	24.522[a]	3	<.001
Likelihood Ratio	24.809	3	<.001
N of Valid Cases	398		

a. 0 cells (0.0%) have expected count less than 5. The minimum expected count is 24.62.

disease * Not wanting to know if something was wrong

Crosstab

			Not wanting to know if something was wrong		Total
				Not wanting to know if something was wrong	
disease	Asthma	Count	110	9	119
		% within disease	92.4%	7.6%	100.0%
		Standardized Residual	.6	-1.5	
	COPD	Count	123	33	156
		% within disease	78.8%	21.2%	100.0%
		Standardized Residual	-1.2	3.1	
	Broch	Count	53	3	56
		% within disease	94.6%	5.4%	100.0%
		Standardized Residual	.6	-1.5	
	ILD	Count	63	4	67
		% within disease	94.0%	6.0%	100.0%
		Standardized Residual	.6	-1.5	
Total		Count	349	49	398
		% within disease	87.7%	12.3%	100.0%

Chi-Square Tests

	Value	df	Asymptotic Significance (2-sided)
Pearson Chi-Square	18.788[a]	3	<.001
Likelihood Ratio	18.513	3	<.001
N of Valid Cases	398		

a. 0 cells (0.0%) have expected count less than 5. The minimum expected count is 6.89.

disease * Not knowing the signs of potential lung disease

Crosstab

			Not knowing the signs of potential lung disease		Total
				Not knowing the signs of potential lung disease	
disease	Asthma	Count	106	13	119
		% within disease	89.1%	10.9%	100.0%
		Standardized Residual	2.0	-3.3	
	COPD	Count	98	58	156
		% within disease	62.8%	37.2%	100.0%
		Standardized Residual	-1.5	2.6	
	Broch	Count	45	11	56
		% within disease	80.4%	19.6%	100.0%
		Standardized Residual	.6	-1.0	
	ILD	Count	43	24	67
		% within disease	64.2%	35.8%	100.0%
		Standardized Residual	-.9	1.5	
Total		Count	292	106	398
		% within disease	73.4%	26.6%	100.0%

Chi-Square Tests

	Value	df	Asymptotic Significance (2-sided)
Pearson Chi-Square	28.203[a]	3	<.001
Likelihood Ratio	30.454	3	<.001
N of Valid Cases	398		

a. 0 cells (0.0%) have expected count less than 5. The minimum expected count is 14.91.

Appendix 3

The questions (7 questions) that have other (others, pleases specify) for an answer

1. Thinking just about your diagnosis of COPD, which of the following symptoms did you experience before seeking professional help. Please tick all that apply

2. What caused you to notice something was wrong? Please tick all that apply

3. What motivated you to seek professional help? Please tick all that apply

4. What, if anything, prevented you from seeking professional help earlier? Please tick all that apply

5. If you feel that your diagnosis was delayed, how did this affect you? Please tick all that apply

6. What factors do you think contributed to your COPD? Please tick all that apply?
